# Efficacy of Liming Forest Soil in the Context of African Swine Fever Virus

**DOI:** 10.3390/v14040734

**Published:** 2022-03-31

**Authors:** Franziska Tanneberger, Ahmed Abd El Wahed, Melina Fischer, Paul Deutschmann, Hanna Roszyk, Tessa Carrau, Sandra Blome, Uwe Truyen

**Affiliations:** 1 Institute of Animal Hygiene and Veterinary Public Health, Faculty of Veterinary Medicine, University of Leipzig, An den Tierkliniken 1, 04103 Leipzig, Germany; franziska.tanneberger@web.de (F.T.); ahmed.abd_el_wahed@uni-leipzig.de (A.A.E.W.); 2 Institute of Diagnostic Virology, Friedrich-Loeffler-Institut, Suedufer 10, Insel Riems, 17493 Greifswald, Germany; melina.fischer@fli.de (M.F.); paul.deutschmann@fli.de (P.D.); hanna.roszyk@fli.de (H.R.); tessa.carraugarreta@fli.de (T.C.); sandra.blome@fli.de (S.B.)

**Keywords:** African swine fever virus, Modified Vaccinia Ankara virus, soil, disinfectant, lime

## Abstract

Since September 2020, Germany has experienced the first ever outbreak of African swine fever (ASF). The first known cases occurred exclusively in wild boar in forest areas in Brandenburg and Saxony; in July 2021, infected domestic pigs were also confirmed for the first time. As wild boar are considered the main reservoir for the virus in the European region, an effective interruption of this infection chain is essential. In particular, the removal and safe disposal of infected carcasses and the direct disinfection of contaminated, unpaved ground are priorities in this regard. For the disinfection, highly potent as well as environmentally compatible disinfectants must be used, which are neither influenced in their effectiveness by the soil condition nor by increased organic contamination. Thus, in this study, slaked lime, milk of lime and quicklime (1% to 10% solutions) were selected for efficacy testing against the test virus recommended by the German Veterinary Society (DVG), Modified Vaccinia Ankara virus (MVAV), and ASF virus (ASFV) in conjunction with six different forest soils from Saxony in two different soil layers (top soil and mineral soil) each. In summary, 10% of any tested lime type is able to inactivate both MVAV and ASFV under conditions of high organic load and independent of the water content of the soil. At least a 4 log reduction of the virus titer in all tested forest soil types and layers and by all applied lime types was observed. In conclusion, the high efficacy and suitability of all tested lime products against both viruses and in the presence of high organic load in forest soil can be confirmed and will help to control ASF spread.

## 1. Introduction

African swine fever virus (ASFV) is one of the most important emerging animal diseases, which spreads rapidly worldwide. From 2007, ASFV spread through Georgia and Russia to the EU in the Baltic states. From there, it continuously spread westwards and in September 2020 the first case in Germany was diagnosed in the state of Brandenburg [1]. Within one year, the cases of the haemorrhagic disease of pigs spread across the states of Brandenburg and Saxony, with more than 2000 cases reported in wild boar one year later [2]. Moreover, three ASF outbreaks were confirmed in Brandenburg domestic pigs in July 2021 [2]. In all cases, local virus variants were also found to be circulated in the wild boar population [3]. Thus, the epidemic in the local wild boar population was the most likely source of these outbreaks. To prevent further spread and economic losses, the German government has implemented strict emergency and hygiene plans in accordance with European legislation (e.g., Commission Implementing Regulation (EU) 2021/605). The control strategy relies on reducing the number of wild boar, fencing of affected areas, monitoring of the susceptible population and proper disposal of infected carcasses [4,5,6]. Furthermore, the disinfection of potentially contaminated soil beneath and around the ASFV-positive carcasses contributed to the active control measures.

The stability of ASFV in the environment is well documented [7,8,9]. ASFV belongs to the family of *Asfarviridae*, which is enveloped and can be easily inactivated by commonly used disinfectants like NaOH or formaldehyde [8]. Peracetic acid and citric acid were shown to be highly effective with ASFV contaminated soil [10], but may be of limited efficacy in the presence of blood [11,12]. Nevertheless, screening the effectiveness of other disinfectants is necessary. Various lime products have been used for decades [13,14,15,16] and are considered to be promising solutions because of their availability in powdery form and their environmentally friendly characteristics. The approval of the use of disinfectants against specific animal diseases in Germany is nationally regulated and linked to an efficacy testing according to test protocols of the German Veterinary Society (DVG). The DVG has recommended the *Orthopoxvirus* Modified Vaccinia Ankara virus (MVAV) as a representative of enveloped viruses [17]. The aim of the study was to test the disinfection potency of lime according to the guidelines of the DVG on MVAV and try to reproduce the results using ASFV.

In order to determine effective concentrations to inactivate enveloped viruses in contaminated soil, various concentrations of slaked lime (Ca(OH)_2_), quicklime (CaO) and lime milk (Ca(OH)_2_ in water) were tested. We have focused on six soil types in one of the most affected states in Germany, Saxony, and followed the guidelines of the DVG of using MVAV for screening the disinfectants. The most effective concentration was then tested on ASFV contaminated soil under appropriate high containment conditions. In addition, the best water/lime ratio was determined. The experimental layout is presented in Table 1.

## 2. Materials and Methods

### 2.1. Study Sites and Soil Types

**Figure 1:**
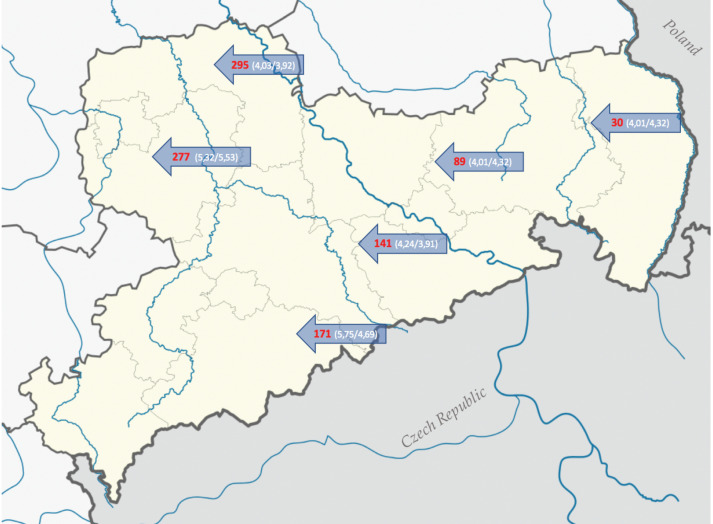
Collection spots of the soil samples. Official names by the authority of Sachsenforst, Pirna, Germany (arrows, red), the numbers in brackets describe the pH of top soil (first number) and mineral soil (second number). Forest stand of soil 277 and 89 is deciduous forest, of 171 and 141 spruce forest and of 295 and 30 pine forest. Map credit [18] (Original Author: TUBS; license link: https://commons.wikimedia.org/wiki/Commons:GNU_Free_Documentation_License,_version_1.2).

**Figure 2:**
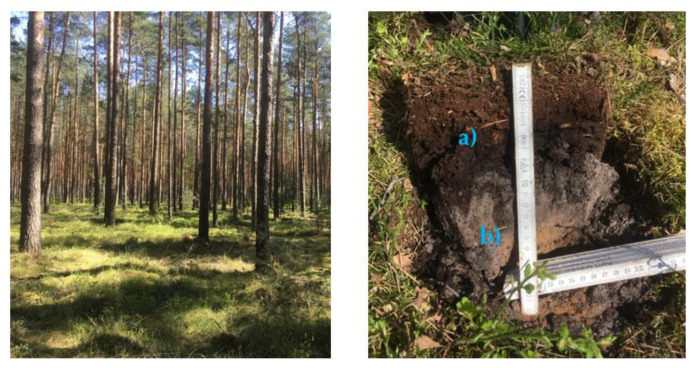
Exemplary representation of a sampled pine forest (**left**) with the top soil (**a**) and upper mineral soil (**b**).

As illustrated in Figure 1, six different soil types across Saxony were selected. For each type, top soil (TS) and mineral soil layer (MS) were collected (Figure 2). For all soil samples, quantitative polymerase chain reaction (real-time PCR) was performed to proof their freedom of both ASFV and MVAV.

### 2.2. Disinfection Experiment with Slaked Lime, Lime Milk and Quicklime (Lime Experiments)

Three different lime products were examined in a suspension form: powdery slaked lime (Chemdiscount/WHC, Hilgertshausen, Germany), powdery quicklime (Carl Roth, Karlsruhe, Germany) and lime milk (made by mixing the slaked lime with water at various concentrations). The lime milk was freshly prepared to a stock solution before using in the experiment, representing 3.4-fold the desired final concentration of either 1%, 5% or 10%. A volume of 2.7 (diameter) + 1.5 (height) cm of TS and MS of each of the 6 soil types corresponding to about 3 mL soil were filled in 50-mL centrifuge tubes, and 3 mL of virus (10^8^ TCID_50_/mL for MVAV or 10^6.75^ TCID_50_/mL for ASFV Armenia ∆258L GFP huCD4 [19]) and 4 mL of fetal calf serum (FCS) were added and mixed for 5 s. To obtain the end concentrations for the powdery disinfectants (slaked lime or powdery quicklime) in the suspension, 0.7 g for 10%, 0.35 g for 5% or 0.07 g for 1% of were added to the mixture of soil, virus and FCS (Supplementary Table S5). In the case of lime milk, 2.9 mL of each of 3.4-fold concentrated lime milk was added to the centrifuge tubes (Supplementary Table S6). As an example, for a final working solution of 1%, 3.4% of the disinfectant was added. Thereafter, the complete mixture was vortexed for 5 s. After incubation at 10 °C for 2 h, ice-cold PBS was added to generate a final virus dilution of 1:4. The mixture was then sonicated in an ultrasound bath (Bandelin Sonorex Super RK 103H, Berlin, Germany) at 4 °C for 5 min and centrifuged at 4500 rpm at 4 °C for 5 min. The supernatant, approximately, 5 mL was collected and filtrated (Filtropour 0.45 µm, Sarstedt, Nuembrecht, Germany). To determine the loss of infectivity, ten-fold dilutions were prepared and cultivated as described previously [10]. To avoid cell toxicity, in some cases, 25 µL was applied to either 24- or 96-well plates.

The experiment for each concentration of the lime products in each soil type and layer were done in duplicates. For each control, the experimental setup remained the same but without the disinfectant. The final virus dilution of the control suspensions at the end of the experiment corresponds to that of the suspensions with disinfectant. Quantitative real-time PCR to determine the total amount of viral DNA for both MVAV and ASFV were done in all disinfection experiments as described previously [10]. This was done to rule out adhesion of infectious virus particles to sand particles, which would have potentially led to false positive disinfection results due to such reduced recoverability. A true disinfection will lead to a decrease in infectivity (virus titer in TCID_50_) at a constant DNA genome copy number concentration.

### 2.3. Lime/Water Ratio

To determine the amount of water needed to activate the powdery lime products (quicklime and slaked lime), various water contents of soil were tested. The same experimental layout for MVAV was performed as above for one MS and one TS with little modifications (Table 2). Briefly, the volume of MVAV was reduced to 1 mL to decrease the water content by using a high titer virus stock. Similarly, FCS was replaced by 0.24 g bovine serum albumin (BSA) in a powder form. The powdery disinfectants were added to the centrifuge tubes containing an equivalent to 3 mL soil in a mass of 0.42 g. After vortexing of the mix for 5 s, one tube was left without adding more water and 420, 840, 1680 and 3360 µL of water with standardized hardness level (WSH) [19,20] was added to reach a ratio of disinfectant of 0 (no further dilution), 1:2; 1:3; 1:5 and 1:9 in the experimental solution. Furthermore, one control tube without disinfectant was prepared (Table 2). The mixture was vortexed again and incubated at 10 °C for 2 h. Subsequently, ice-cold PBS was added to give a final virus dilution of 1:8 in the tubes to stop the disinfectant activity. The supernatant was collected and immediately inoculated into the cell culture system as described previously [10]. DNA extraction and real-time PCR was performed for samples as published previously [10]. Each experiment was performed twice.

### 2.4. Avoidance of Cell Toxicity

The experimental use of highly potent disinfectants in conjunction with cell cultures can lead to pronounced cell-toxic effects that can severely compromise the validity of results, especially for the exclusive evaluation via cytopathogenic effects (CPE) like in the case of MVA.

While the CPE of MVA appears in the form of swollen, blistered and rounded cell bodies, toxic effects present themselves in the form of black staining (necrosis) and detachment of the cells. Only in case of high-grade cell toxicity, no more healthy cells remain to identify the CPE, which would make correct evaluation impossible.

Extensive preliminary tests were carried out to investigate the toxicity of the different types and amounts of lime on the corresponding cell cultures.

For this purpose, the respective lime concentrations (1%, 5%, 10%) in the final dilutions used in the experiment were titrated without virus and added to the corresponding cell culture in 96-well plates. The cell culture was incubated and tested for toxicity over 2 h. If cell toxicity (necrosis/detachment of cells) was observed during this time in one or more dilution levels, the respective dilution level was added in addition to a 24-well plate containing cell culture. Again, incubation and observation of toxicity was performed for 2 h. If toxicity was no longer detectable here in the respective dilutions, inoculation of the critical dilution levels could be performed in larger cell culture plates in the main experiment (containing disinfectant and virus). Throughout the experiments, at least one control was titrated per experiment (tube with regular experimental setup and virus but without addition of disinfectant) to ensure a visual comparison between potential cell toxicity and pure CPE. This allowed optimal discrimination between toxic effects and CPE.

## 3. Results

### 3.1. Disinfection of MVAV and ASFV with Lime (Lime Experiments)

**Figure 3:**
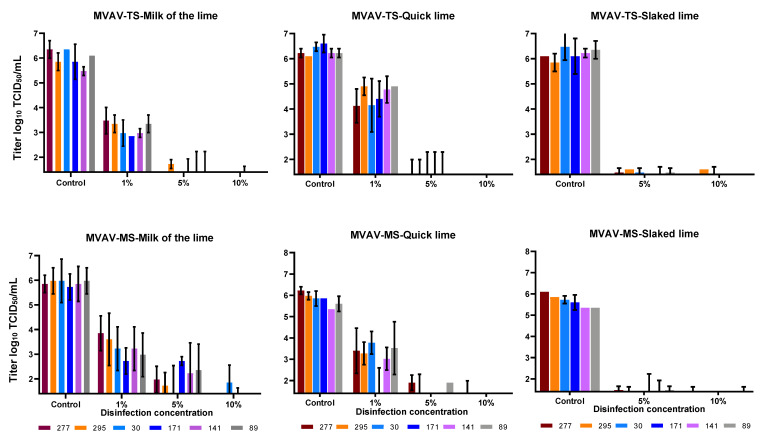
Comparison of the disinfection of MVAV with three different types of lime. Top three panels represent soil (TS) and lower three panels mineral soil (MS). The mean value and standard deviation of at least duplicate tests are shown. Virus titers were calculated by Spearman–Kaerber method. Limit of detection was 1.4 log_10_ TCID_50_/mL.

**Figure 4:**
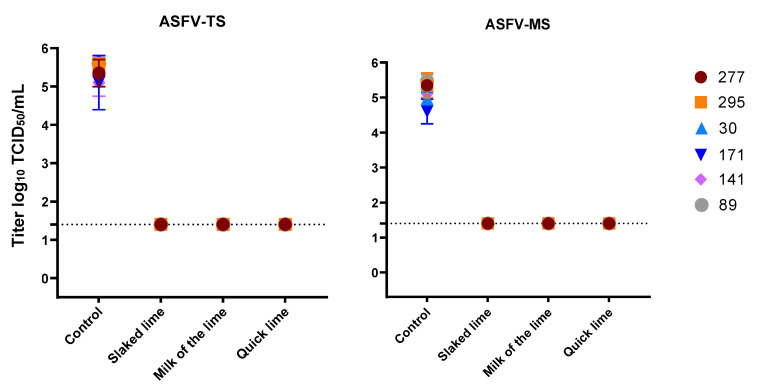
Disinfection of ASFV with three different types of lime in a concentration of 10%. The mean value and standard deviation of at least duplicate tests are shown. Virus titers were calculated by Spearman–Kaerber method. Limit of detection was 1.4 log_10_ TCID_50_/mL.

Various types and concentrations of lime were used to deactivate MVAV in the presence of high organic contents (FCS). For lime milk, quicklime and slaked lime, 10% was sufficient to reduce the viral titer of MVAV by at least 4 logs (Figure 3). Same efficacy was seen against ASFV, as a concentration 10% of all lime types revealed a complete inactivation of ASFV in all soil types tested (Figure 4). The viral load as determined by real-time PCR for both the control and the experimental set revealed similar values (Supplementary Tables S1–S3).

### 3.2. Lime/Water Ratio

The influence of water contents on the disinfectant’s potency was tested. A complete inactivation was observed, when no extra water or up to 16 times the volume of the lime powder extra water was added. The amount of added water had no influence on the inactivation of MVAV in soil (Table 2). The viral load as determined by real-time PCR for both the control and the experimental set (Supplementary Table S4 behaved similar to the viral loads of the lime experiments).

## 4. Discussion

Decontamination of soil beneath and around carcasses can play an important role in preventing spread of ASFV. Many studies were conducted on the efficacy of the disinfectants on contaminated solid floor and walls of stables [21,22,23], but few have been performed on forest soil, which is considered to contribute to the spread of the virus within the wild boar population in Germany. The World Organization for Animal Health (OIE) and the German Federal Ministry of Food and Agriculture (BMEL) recommended the removal of the soil beneath the carcasses under biosecurity measurements [6,24,25,26]. To avoid further spread of ASFV during handling and transportation of infected soils, the easier and quicker alternative is an effective soil disinfection onsite. In this study, the virucidal effectivity of slaked lime, quicklime and lime milk were examined. The disinfectants were tested first with MVAV as recommended by DVG on six soil types under BSL-2 conditions and applied to the ASFV in a high-containment facilities. At least a 4 log_10_ TCID_50_/mL inactivation was achieved with 10% for all three tested lime types at 10 °C and an exposure time of two hours. To simulate a field situation, the experiments were done with high organic soiling. In a previous study, 0.1% peracetic acid completely inactivated ASFV in various soil types, while citric acid had only limited efficacy [10]. While blood may reduce the efficacy on peracetic acid performance [11], lime was shown to be effective in in the presence of blood, decomposition material or other disruptive substances [27]. Powdery slaked lime and powdery quicklime are commonly used for the disinfection of poultry farms in case of avian influenza virus [28,29]. Lime milk is the disinfectant of choice in the elimination of the infectivity of contaminated slurry [30,31] or ponds [14,31].

The powder form of lime has an advantage of ease of transportation to the affected area and to the target zone in forest, where carcasses are found. Ambient temperature has little to no effect on lime decontamination characteristics [27]. Less or extensive water contents of the matrix can reduce the virucidal properties of powdery lime products [32]. In contrast, in our experiment, water contents did not influence the inactivation of MVAV in soil by powdery lime. Our experiment was designed to reduce the water content of the mixtures as much as possible by using dry soil, BSA in powder form, and a high virus stock in a small volume). However, our study was performed on different soils collected from Saxony, Germany, from areas with varying annual rainfall (656–1146 mm). Therefore, it is highly recommended to test the system before widespread application in a particular environment, country or soil type.

The mechanism behind this class of disinfectants is the alkalization of the matrix (pH up to 12 or higher) [31,33]. This pH level causes the denaturation and coagulation of proteins [31]. Despite the high acid content of soil in Saxony, Germany [10], the pH of the soil after adding the lime was raised to pH 11 to 12 [34]. Furthermore, in the case of quicklime, exothermal reaction with water with a possible heating up to 80 °C will enforce the microbiocidal potency [31]. However, the increased risk of forest fires must be taken into account here, especially in summer. Additionally, special precautions to protect the health of workers must be taken to prevent the potential caustic effect on skin, eyes, lungs and exposed mucosa [31].

DVG in Germany has recommended the MVAV as a representative for enveloped viruses for testing the efficacy of disinfection solutions [17]. A previous study, comparing the efficacy of peracetic and citric acids to inactivate both MVAV and ASFV, has shown only little inconsistency between both viruses [10]. In the experiments described here with the lime products, both viruses are once again showing a very similar behaviour during disinfection under mentioned conditions. Compared to peracetic acid and citric acid, lime is not affected by low ambient temperature or any kind of organic load. This fact makes lime a highly efficient as well as simultaneously cost-effective means of combating ASF.

## 5. Conclusions

In conclusion, lime (quicklime, slaked lime, lime milk) are effective for the disinfection of forest soil contaminated with ASFV, especially in the presence of high organic soiling.

## Figures and Tables

**Table 1:** Experimental layout.

Virus	BSL	Disinfection
MVAV	BSL-2	1%, 5%, 10% slaked lime 1%, 5%, 10% quicklime 1%, 5%, 10% lime milk
ASFV Armenia ∆258L GFP huCD4	BSL-4	10% slaked lime 10% quicklime 10% lime milk

**Table 2:** Setup and results of lime/water ratio experiments. WSH (water with standardized hardness level) was used in the experiments. The water content has no influence on the efficacy of the liming. No infectious virus was detected after disinfection in any soil type, independent of the water content. The limit of detection is 1.7 Log_10_ TCID_50_/mL. Control value is the average of virus concentrations in TS and MS of 277.

Ratio	Soil (mL)	MVAV (mL)	BSA (g)	Slaked Lime or Quicklime (g)	WSH (µL)	PBS (Reaction Stop) (mL)	Total Virus Dilution	Virus Titer (Log_10_ TCID_50_/mL)
0	3	1	0.24	0.42	0	7	1:8	ND
1:2					420	6.58		ND
1:3					840	6.16		ND
1:5					1680	5.32		ND
1:9					3360	3.64		ND
Control				0	0	7		6.04

## Data Availability

All data are available in the manuscript and the Supplementary Materials.

## Supplementary Materials

The following supporting information can be downloaded at: https://www.mdpi.com/article/10.3390/v14040734/s1. The Supplementary File contains the real-time PCR results. Table S1: qPCR—lime experiments—**MVA**—Of (top soil) of soil samples 277, 295, 30, 171, 141, 89; Table S2: qPCR—lime experiments—**MVA**—A (mineral soil) of soil samples 277, 295, 30, 171, 141, 89; Table S3: qPCR—**ASFV**—lime experiments—Of and A (top soil and mineral soil) of soil samples 277, 295, 30, 171, 141, 89; Table S4: qPCR—lime/water-ratio—**MVA**—Of and A (top soil and mineral soil) of soil sample 277; Table S5: Final concentrations and added amount of quicklime and slaked lime in experimental layout for lime experiments; Table S6: Stock dilution and final concentrations of lime milk in experimental layout for lime experiments.
