# Supplementary material for: Efficacy of Liming Forest Soil in the Context of African Swine Fever Virus
Source: Viruses. 2022 Mar 31;14(4):734. doi: 10.3390/v14040734 (PMC9025520; doi:10.3390/v14040734)
Supplement: Supplementary file 1 [file viruses-14-00734-s001.zip › viruses-1579926-supplementary.pdf]

Supplementary Table S1 – qPCR – lime experiments (as explained in section 2.2) - **MVA** – Of (top soil) of soil samples 277, 295, 30, 171, 141, 89

	**277 - Of**		**295 - Of**		**30 - Of**		**171 - Of**		**141 - Of**		**89 - Of**	
	cT	copies	cT	copies	cT	copies	cT	copies	cT	copies	cT	copies
**Slaked lime**	28,475	4,01E+03	29,57	1,99E+03	31,265	1,12E+03	26,4	9,58E+03	29,005	6,60E+03	29,5	1,58E+03
**Lime milk**	30,835	1,34E+03	31,425	3,83E+03	31,25	1,72E+03	31,035	2,28E+03	31,625	1,09E+03	33,775	9,50E+02
**Quicklime**	30,22	4,93E+02	29,46	1,01E+03	31,755	1,92E+02	29,705	1,34E+03	31,75	3,40E+02	31,585	2,37E+02

	**277 - Of – Contr.**		**295 - Of – Contr.**		**30 - Of – Contr.**		**171 - Of – Contr.**		**141 - Of – Contr.**		**89 - Of – Contr.**		**MVA + PBS**	
	cT	copies	cT	copies	cT	copies	cT	copies	cT	copies	cT	copies	cT	copies
**Slaked lime – experim.**	23,9	3,54E+04	23,05	5,95E+04	23,4	5,17E+04	25,99	1,18E+04	24,44	2,55E+04	24,54	2,41E+04	23,275	5,78E+04
**Lime milk – experim.**	28,58	5,01E+03	24,75	4,15E+04	26,07	2,59E+04	24,01	6,05E+04	27,83	9,00E+03	23,64	7,28E+04	21,7	2,42E+05
**Quicklime – experim.**	23,29	4,42E+04	22,79	6,12E+04	22,32	8,94E+04	24,3	2,53E+04	24,47	2,32E+04	24,46	2,23E+04	23,125	5,45E+04

Supplementary Table S2 – qPCR – lime experiments (as explained in section 2.2) - **MVA** – A (mineral soil) of soil samples 277, 295, 30, 171, 141, 89

	**277 - A**		**295 - A**		**30 - A**		**171 - A**		**141 - A**		**89 - A**	
	cT	copies	cT	copies	cT	copies	cT	copies	cT	copies	cT	copies
**Slaked lime**	31,585	3,31E+02	29,555	1,27E+03	29,515	1,17E+03	29,31	1,33E+03	29,01	1,70E+03	30,93	8,91E+02
**Lime milk**	31,755	1,16E+03	32,73	9,96E+02	32,095	1,79E+03	33,335	5,05E+02	33,205	5,26E+02	31,11	1,63E+03
**Quicklime**	30,55	3,97E+02	30,62	5,60E+02	30,085	6,46E+02	31,865	2,60E+02	31,32	5,35E+02	30,48	4,27E+02

	**277 - A – Contr.**		**295 - A – Contr.**		**30 - A – Contr.**		**171 - A – Contr.**		**141 - A – Contr.**		**89 - A – Contr.**		**MVA + PBS**	
	cT	copies	cT	copies	cT	copies	cT	copies	cT	copies	cT	copies	cT	copies
**Slaked lime – experim.**	24,095	4,65E+04	27,7	4,46E+03	28,47	9,33E+02	26,165	5,14E+03	34,38	3,42E+03	38,53	3,31E+04	22,86	3,78E+04
**Lime milk – experim.**	26,22	2,34E+04	26,69	9,58E+03	27,62	1,35E+04	28,275	8,05E+03	28,25	1,02E+04	38,495	1,47E+03	22,55	1,67E+05
**Quicklime – experim.**	23,165	4,87E+04	38,9	1,60E+03	31,825	2,23E+02	26,535	6,02E+03	28,185	4,87E+03	29,655	2,55E+03	23,275	4,71E+04

Supplementary Table S3 – qPCR – **ASFV** – lime experiments (as explained in section 2.2) - Of and A (top soil and mineral soil) of soil samples 277, 295, 30, 171, 141, 89

	**277 - Of**		**295 - Of**		**30 - Of**		**171 - Of**		**141 - Of**		**89 - Of**		**ASFV + PBS**	
	cT	copies	cT	copies	cT	copies	cT	copies	cT	copies	cT	copies	cT	copies
**Slaked lime**	25,42	1,44E+02	23,675	4,62E+02	24,705	2,41E+02	24,355	2,93E+02	23,845	4,24E+02	25,745	1,26E+02	-	-
**Lime milk**	24,3	3,14E+02	23,29	6,05E+02	23,125	6,75E+02	23,27	6,18E+02	24,12	3,94E+02	24,61	2,75E+02	-	-
**Quicklime**	25,865	1,05E+02	25,01	1,89E+02	25,91	1,04E+02	25,435	1,41E+02	24,665	1,66E+02	25,83	1,08E+02	-	-
**Control**	24,12	3,42E+02	23,12	6,92E+02	22,82	8,27E+02	24,11	3,45E+02	23,72	4,53E+02	23,06	7,05E+02	22,9	7,88E+02

	**277 - A**		**295 - A**		**30 - A**		**171 - A**		**141 - A**		**89 - A**		**ASFV + PBS**	
	cT	copies	cT	copies	cT	copies	cT	copies	cT	copies	cT	copies	cT	copies
**Slaked lime**	27,955	3,53E+01	27,74	3,42E+01	27,91	3,97E+01	30,195	6,50E+00	29,175	1,64E+01	29,605	1,13E+01	-	-
**Lime milk**	27,445	8,28E+01	26,6	9,50E+01	26,06	1,21E+02	30,065	9,30E+00	27,055	6,23E+01	27,61	4,67E+01	-	-
**Quicklime**	28,715	2,99E+01	27,435	4,09E+01	27,23	5,85E+01	30,145	1,02E+01	29,005	2,21E+01	29,4	1,68E+01	-	-
**Control**	23,62	5,18E+02	24,69	2,58E+02	24,01	4,02E+02	26,33	8,62E+01	25,16	1,88E+02	25,5	1,49E+02	23,08	8,32E+02

Supplemenatry Table S4 – qPCR – lime/water-ratio (as explained in section 2.3) - **MVA** – Of and A (top soil and mineral soil) of soil sample 277

	**0**		**1:2**		**1:3**		**1:5**		**1:9**		**MVA + PBS (control)**	
	cT	copies	cT	copies	cT	copies	cT	copies	cT	copies	cT	copies
**Slaked lime - Of**	35,99	9,96E+00	34,79	3,14E+01	32,525	9,84E+01	30,495	4,09E+02	31,1	2,96E+02	25,855	8,58E+03
**Quicklime-Of**	35,64	3,05E+01	33,575	4,57E+01	30,31	6,89E+02	29,015	1,35E+03	31,97	2,41E+02	24,14	5,01E+04

	**0**		**1:2**		**1:3**		**1:5**		**1:9**		**MVA + PBS (control)**	
	cT	copies	cT	copies	cT	copies	cT	copies	cT	copies	cT	copies
**Slaked lime - A**	32,825	1,76E+02	33,22	1,38E+02	35,215	2,89E+01	33,665	4,75E+01	32,39	1,90E+02	26,47	5,61E+03
**Quicklime - A**	32,35	1,09E+02	33,3	1,68E+02	32,915	2,48E+02	30,62	4,65E+02	31,795	1,78E+02	25,935	1,46E+04

Supplementary Table S5– Final concentrations and added amount of quick lime and slaked lime in experimental layout for lime experiments (see 2.2 in manuscript

**Disinfectant**	**Virus (mL)**	**FCS (mL)**	**Amount of disinfectant added (g)**	**Concentration during experiment**
Quick lime/ Slaked lime (powder)	3	4	0.07	1%
			0.35	5%
			0.7	10%

Supplementary Table S6 – Stock dilution and final concentrations of lime milk in experimental layout for lime experiments (see 2.2 in manuscript); each stock was freshly prepared with WSH (water with standardized hardness level) directly before each experiment

**Disinfectant**	**Virus (mL)**	**FCS (mL)**	**Stock concentration** (x3.4 of desired concentration)	**Added volume of stock (mL)**	**Concentration during experiment**
Lime milk (solution)	3	4	3,4%	2.9	1%
			17%	2.9	5%
			34%	2.9	10%
